# Global donkey and mule populations: Figures and trends

**DOI:** 10.1371/journal.pone.0247830

**Published:** 2021-02-25

**Authors:** Stuart L. Norris, Holly A. Little, Joseph Ryding, Zoe Raw

**Affiliations:** The Donkey Sanctuary, Sidmouth, Devon, United Kingdom; Sejong University, REPUBLIC OF KOREA

## Abstract

Knowing how many donkeys there are in specific countries where welfare is compromised is a key concern for targeting efforts to improve donkey welfare. Additionally, accurate population estimates are vital for providing evidence and addressing the impact of population threats. The FAO annually report the number of donkeys and mules in each country. The last paper to investigate global and region trends dates back to 2000 and used FAO data from 1961 to 1997. This paper is an update focusing on global, regional and country level donkey and mule populations to understand if there have been any changes in the trends reported by the previous study between 1997 and 2018. Results show that the general trend identified between 1961 and 1997 is continuing with the number of donkeys globally increasing at a rate of ~1% per annum whilst mule populations are in decline at a rate of ~2% per annum. Results also suggest that the trend identified in the original paper are still evident today with the largest increases in donkey population seen in the sub-Saharan African region and greatest reduction noted in Eastern Europe with these two regions having different socio-economic drivers influencing these changes. These results highlight the multifaceted socio-economic drivers influence changes in donkey and mule populations demonstrating the complexity of designing targeted one-welfare approaches. Whilst the FAO donkey and mule datasets are the best available for understanding spatial-temporal distributions in populations there needs to be greater effort to promote the communication of information from the country level to the FAO. This can be directly supported by NGO’s by promoting the robustness of the FAO process for collating and disseminating this information. NGO’s should also seek to highlight the importance of this information for understanding global regional and country level drivers for equid population changes and potential threats to welfare as well as using this information to facilitate projects that support one-welfare approaches.

## Introduction

Working equids support some of the poorest communities in the world, enabling people in low- and middle-income countries to make a living and support their families [1–3]. Working equids are engaged in a wide variety of roles [4–7], and it is widely accepted that improving the welfare of working equids also provides benefits for their owners, as a healthier working animal is able to work more efficiently and provide enhanced income-earning potential [8–10]. Equids also provide many non-working roles such as producing agricultural products such as meat and millk [11], nutraceuticals [12], cosmetics [13], silviculture [14], tourism [15], and onotherapy [16]. A number of non-government organisations (NGOs) work internationally to improve the health and welfare of working and non-working equids, but there is little information published about even some of the most basic aspects of working in this field [17]. One of the major data deficiencies currently surrounds estimations available for global equid population size and distribution. It is often reported that there are an estimated 112 million working equids in the world which support the lives of approximately 600 million people [17–19] but this figure is likely to be a gross underestimate [20] and there is reporting variation regarding what species this figure includes.

In the conservation and management of wild species, it is widely accepted that understanding and documenting species’ population sizes is a central and critical component of successful preservation of biodiversity [21, 22]. Documenting population sizes and distributions is a defining and central concept of the IUCN (International Union for Conservation Nature) Red List of Threatened Species, which is the world’s most comprehensive resource of population data and trends for millions of species. It is based upon a series of ‘species assessments’, whereby technical experts analyse population data for a particular species, and according to a detailed set of guidelines, assign one of eight classifications to that species [23]. The IUCN Red List acts as the central point of reference for any scientist interested in understanding the global conservation status of a species, and provides a succinct analysis of the current population size, past and projected trends. It is a formidable resource for wild species, but makes little contribution to understanding the population dynamics of domesticated or companion species, and expressly excludes domesticated taxa and hybrids [24]. There are six equid (family *Equidae*; genus *Equus*) species listed by the IUCN: Asiatic wild ass *Equus hemionus* (Near Threatened); African wild ass *Equus africanus* (Critically Endangered); Grevy’s zebra *Equus grevyi* (Stable); Plain’s zebra *Equus quagga* (Near Threatened); Przewalski’s Horse *Equus ferus* (Endangered) and the Kiang *Equus kiang* (Least Concern). These are all wild species of equid, and do not include any domesticated species.

Domestic species of *Equus* are horses *Equus caballus*, donkeys *Equus asinus*, and horse-donkey hybrids also known as mules *Equus asinus* × *Equus caballus* [25]. The accepted source of population data for these species in a global context is FAOSTAT [26], the data service maintained by the Food and Agriculture Organization (FAO) of the United Nations (UN). FAOSTAT provides free access to datasets concerning food and agricultural data from around the world, from 1961 to present. Consequently, in terms of domestic animal population figures, this resource focuses mostly on those species which are typically found in live animal production, for example cattle, goats, sheep, pigs, etc., but it also lists horses, asses and mules. The assess and mules categories is described by the FAO as relating to donkeys and mules repetitively, but the former is likely that it also includes mules due to their similarity to donkeys, and probable lack of distinction from donkeys during any population census events and vice versa.

Donkeys and mules are not regarded as being as economically important as other livestock species primarily bred to produce consumable products [2]. FAOSTAT data may therefore not be as reliable for donkeys and mules as it is for other livestock species. In addition, the FAOSTAT dataset for Donkeys does not provide further information on the age of animals, sex ratio, or the purpose for which they are raised, making it difficult to discern the function of the equids, e.g: working, not working, production animal, etc. The FAO also does not provide a breakdown for sub-species or breeds of donkey making it difficult to draw conclusion relating to the relative rates of endangered/extinction risk of a certain populations, as such modeling demographic structure of sub-species or breeds plays a key role in understanding threats to these populations [27].

Nonetheless, the FAOSTAT database is the most reliable source for understanding the size and distribution for global populations of donkeys and mules. A useful summary of global donkey and mule population data was published in 2000, and addressed trends from trends between 1961 and 1996 [2]. Since then, a further twenty-two years of population data are available (1997 to 2018). Whilst the absence of data from the FAOSTAT dataset limits the robustness of this study in the absence of a more complete dataset, We aim to analyse these new data for donkey and mule populations, using the ‘Asses’ and ‘Mules’ data from FAOSTAT [26]. Using this information, we aim to understand population trends were between 1997 and 2018. We present a discussion of global donkey and mule population data trends, and explore how it can be interpreted and utilized by those NGOs working in the international equid welfare sector.

## Materials and methods

### Data sources and collation

The FAO has been publishing Annual Production Yearbooks on live animal stocks since 1961. FAO donkey and mule population trends were obtained from the FAOSTAT [26] website for the years 1961 to 2018 in November 2020. Data were obtained through the live animals FAO database which collates population data on a country by country basis per year where available. The FAO data on livestock numbers are intended to cover all domestic animals irrespective of their age and the place or purpose of their breeding. Estimates are made for non-reporting countries as well as for countries reporting incomplete data. However, for certain countries where no data has ever been reported, such as the United Kingdom, data remains absent.

The FAO official data originates from a government source within each country and are the most reliable FAO figures, however as these originate from countries there maybe undisclosed bias as in part, the donkey is not a productive species and/or because of the specific geopolitical circumstances. Other issues relating to official figures provided by countries to the FAO stem from the numbers may not be accurate due to animals not being officially registered or reported. For example in Europe, many animals may not be registered [11]. Despite this, the FAO attempts to correct this by using estimates despite this there may be historic lack of inaccurate reporting which may impact of the robustness of the data provided by the FAO. In instances where no official data are available, data from semi-official sources (including commodity-specific trade publications such as UN ComTrade [28]) may be used and reported as unofficial figures. These unofficial data sources are still subject to checks by the FAO. If no data from either official or unofficial sources are available but there is data available from surrounding countries the FAO impute data using a 4-level hierarchical linear model.

The FAO uses modelling techniques to impute missing data when there is an absence of official or unofficial data for each member state, however if there is no official data for surrounding countries or proxies measures available within country then data remains absent. The FAO’s imputation method uses observed data from member states and applies cluster analysis to identify relatively homogeneous groups of cases based on selected characteristics, so that variation within groups is minimized and variation between groups is maximized. An explorative hierarchical cluster analysis is first used to visualize similarities among the variables used, followed by K-means clustering, which is used to create the clusters and assign cluster values to each case. The squared Euclidean distance is then chosen as the proximity measure, and representative clusters are identified using the final cluster centers, which represent the average value on all clustering variables of each cluster’s member, and the Euclidean distance between final cluster centers. The clusters obtained are then mapped and characterised in terms of a number of environmental and demographic variables, including poverty estimates. Furthermore, they are compared directly with official data using a correspondence analysis.

### Statistical methods

All statistical analyses were performed using R v3.6.1 [29] and RStudio v1.2 [30]. All percentage and summary calculations were carried out using the R package tidyverse [31]. T-tests, Pearson pair correlations and correlation significance testing between changes in donkey and mule population were carried out using the R package Stats [29] after ensuring data conformed to normality assumptions using Shapiro-Wilk test of normality [29] and plotting the empirical quantiles of each variable using the R package car [32]. As the data conformed to normality assumption no transformation were performed before parametric test were carried out. Choropleth maps of FAO reported 2018 population number for donkey and mules were created by compiling the data as two shapefiles in R using the package sf [33] and then plotted using QGIS [34].

## Results

**Fig 1:**
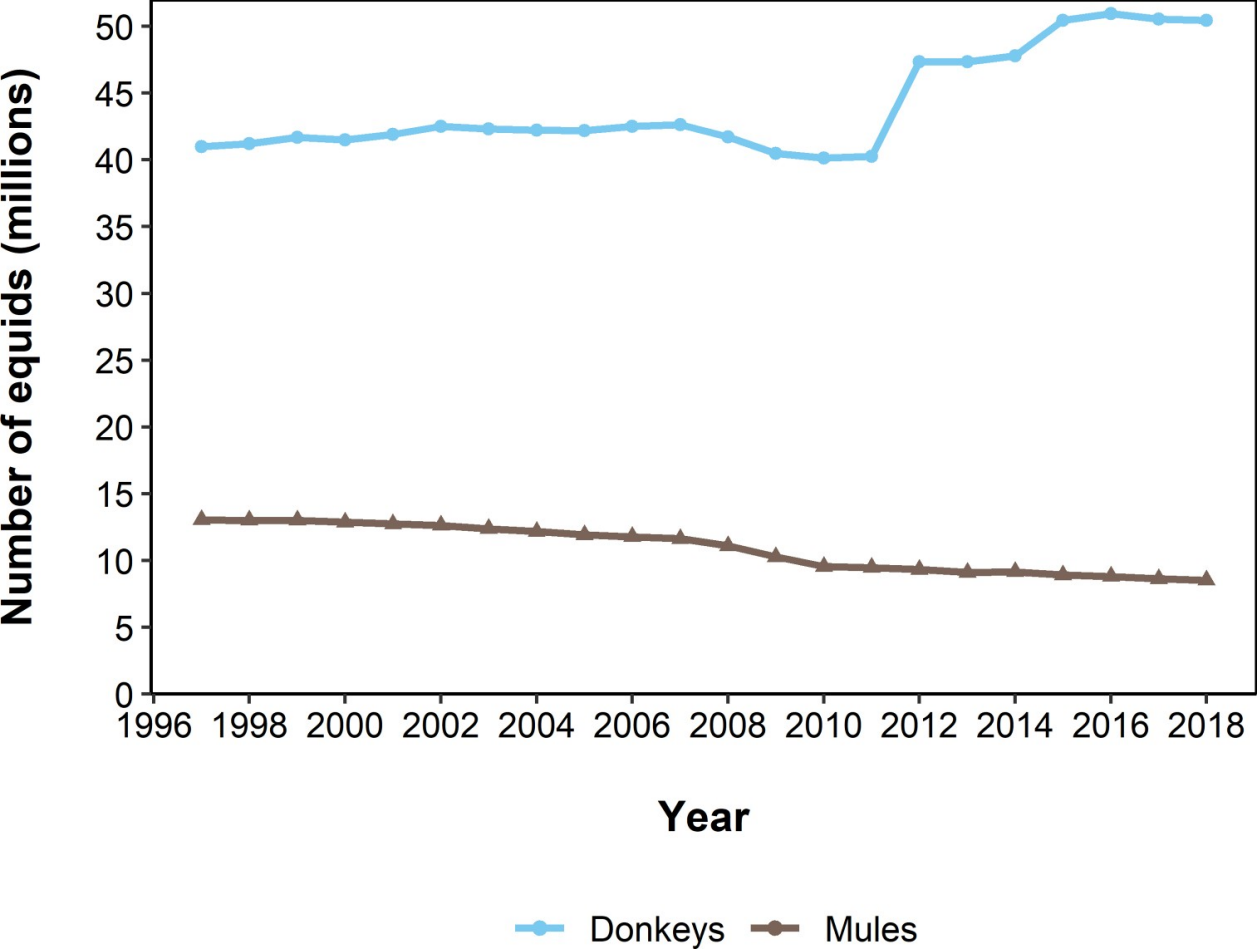
Global population trend of donkeys and mules. Data from FAOSTAT. The blue line and circles represent donkey population trends, the brown line and triangles represent mule population trends.

Since 1997, there has been a 19% increase in the number of donkeys globally from 40,981,873 to 50,451,887. However, there has been a 53% decrease in the global mule population size from 13,050,106 to 8,522,982 (Fig 1). There has been a steady decrease in the number of mules globally (yearly mean = -2%), in contrast the mean percentage change in donkey global donkey population size was 1% with the largest increase in donkey population size in 2012 where there was a 15% increase from 40,277,686 to 47,355,459 and the largest decrease in 2009 (41,703,663, 40,494,756, -3%). This is supported by the overall variance for global donkey population being greater than the variance of global mule populations (σ^2^ = 12.7, σ^2^ = 4.8 respectively) with a weak non-significant correlation between the percentage changes in population size of donkeys and mules (r^2^ = 0.27, *P* = 0.23) indicating that changes in global donkey and mule population size are not linked.

**Fig 2:**
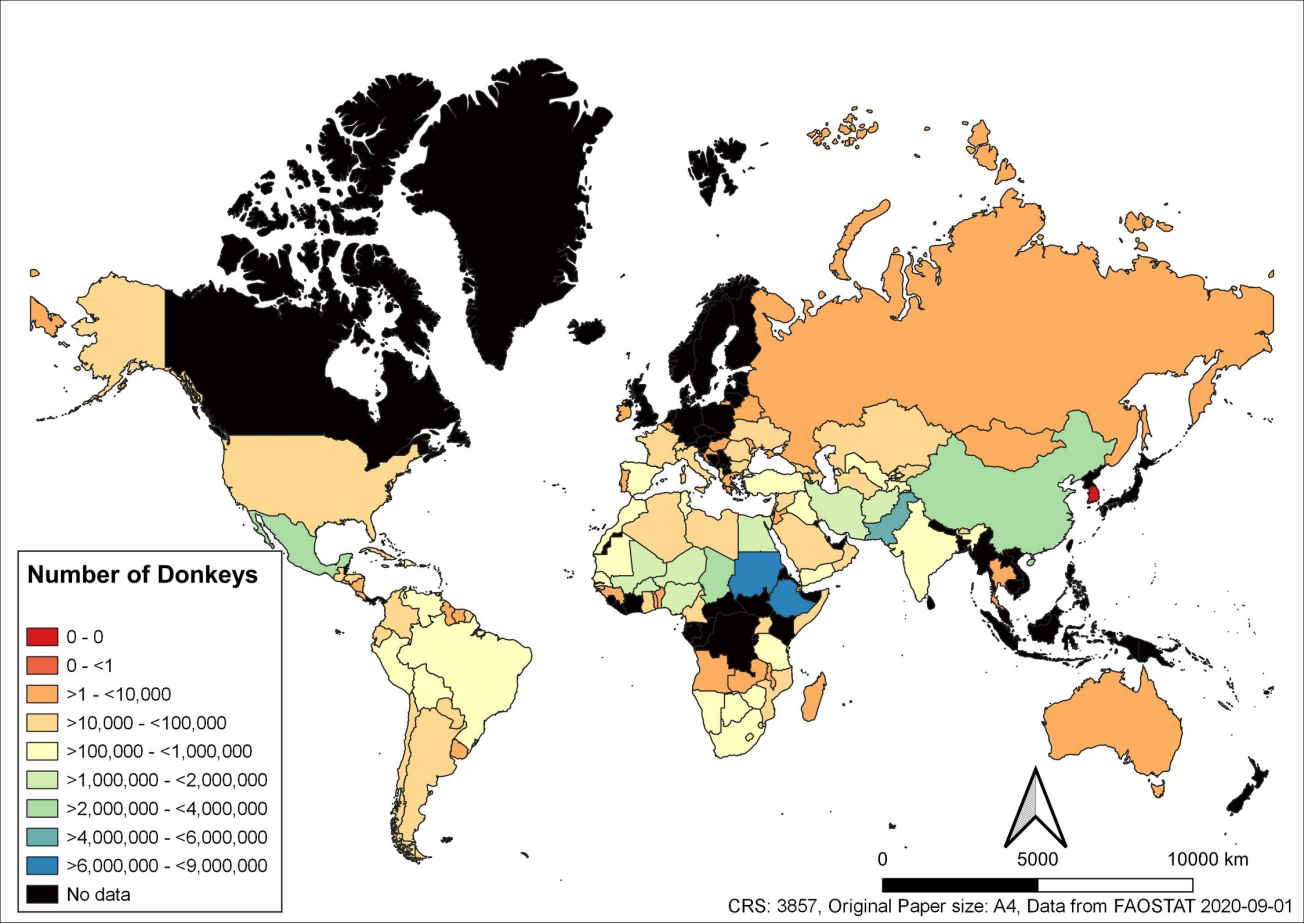
Global donkey population sizes for each country in 2018. Data from FAOSTAT, where data has not been made available to FAOSTAT countries are shaded black.

**Fig 3:**
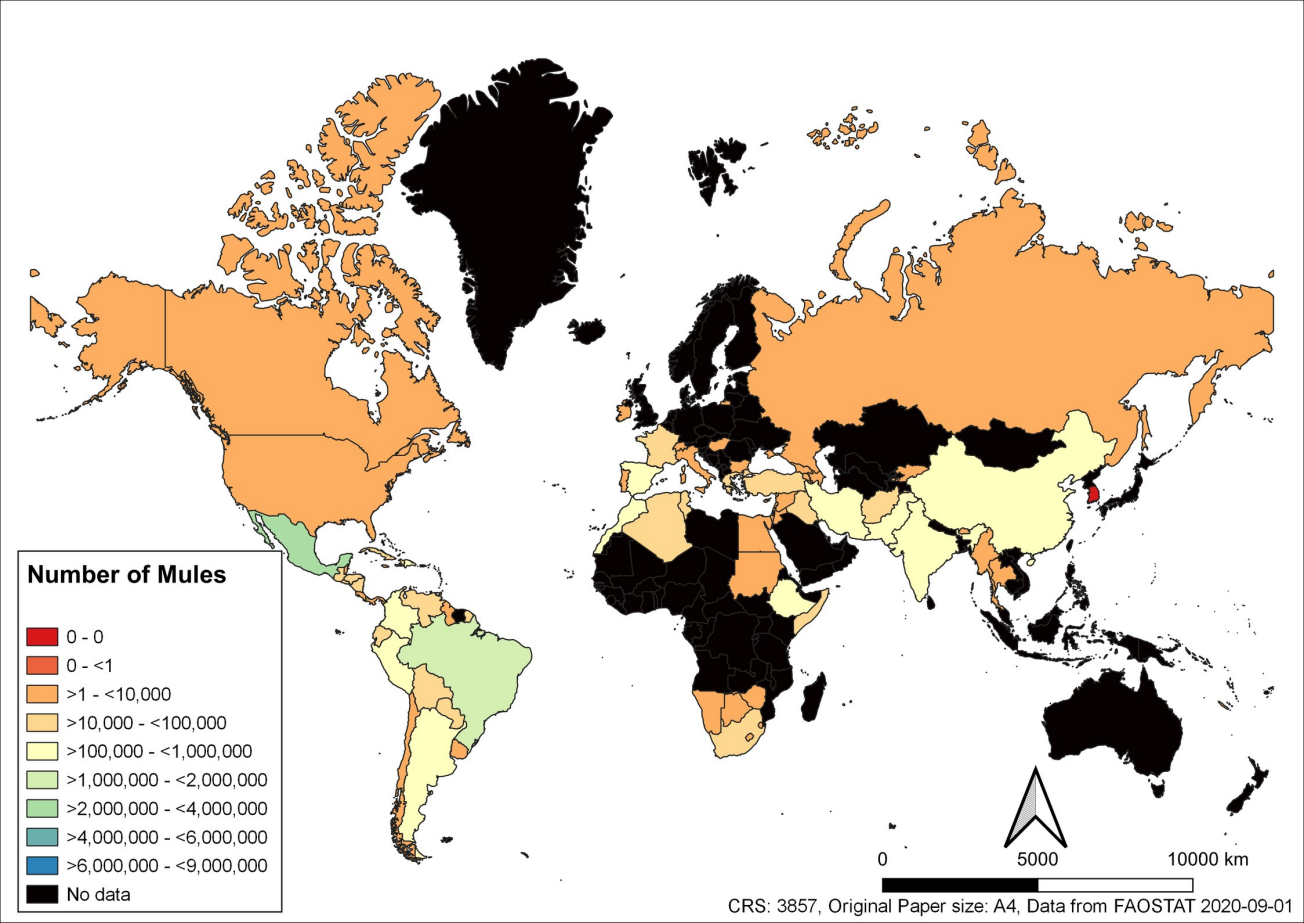
Global mule population sizes for each country in 2018. Data from FAOSTAT, where data has not been made available to FAOSTAT countries are shaded black.

The country with the largest population of donkeys in 2018 was Ethiopia with 8,542,747 donkeys (Fig 2). However, in 1997 the country with the largest population of donkeys was China with 9,444,000 donkeys. The largest population of mules in 2018 was found in Mexico (3,287,449) (Fig 3), similar to donkeys the largest population of mules in 1997 was found in China (4,780,000).

At the country level, the population of donkeys and mules changed between 1997 and 2018 shows that Sudan, Chad and Zimbabwe’s percentage of donkey populations increased by over 80% with Sudan having the largest percentage population change (Table 1A). In contrast, the countries with the greatest reduction in donkey population size were Bulgaria, Greece and Ecuador. Whilst China had the largest change in the number of donkeys between 1997 (9,444,000) and 2018 (2,677,800) the percentage change in herd size was less than that of India with a percentage reduction of 2.5 fold in population size (Table 1A).

**Table 1:** Countries with the 10 greatest increases and reductions in donkey (A) and mule (B) population size between 1997 and 2018 where the population in 2017 was greater than 2500.

A) Donkey Population				B) Mule Population			
Country	1997	2018	Percentage Change	Country	1997	2018	Percentage Change
Sudan	700,000	7,608,854	90.8	France	14,070	27,988	49.7
Chad	341,576	3,080,235	88.9	Ethiopia	240,000	340,358	29.5
Zimbabwe	104,000	585,048	82.2	Peru	224,000	317,664	29.5
Switzerland	9,038	34,028	73.4	Botswana	2,500	3,397	26.4
Cuba	6,200	17,400	64.4	Pakistan	142,000	192,000	26
Ethiopia	3,150,000	8,542,747	63.1	Iran	147,150	178,964	17.8
Mozambique	21,000	49,428	57.5	Somalia	20,000	22,141	9.7
Gambia	32,734	63,781	48.7	Uruguay	3,700	4,080	9.3
Burkina Faso	643,689	1,230,042	47.7	South Africa	14,000	15,291	8.4
Namibia	85,188	153,126	44.4	Dominican Republic	135,000	145,367	7.1
Bulgaria	286,874	19,000	-1409.9	USA	28,000	295	-9391.5
Greece	77,847	8,547	-810.8	Bulgaria	17,432	1,411	-1135.4
Ecuador	267,000	47,035	-467.7	Syria	18,000	2,450	-634.7
Portugal	44,757	7,884	-467.7	Portugal	18,448	3,078	-499.4
Turkey	689,000	141,375	-387.4	China	4,780,000	811,200	-489.3
Colombia	450,000	97,545	-361.3	Turkey	154,000	34,360	-348.2
Armenia	6,837	1,758	-288.9	Algeria	68,740	16,808	-309
India	882,000	229,296	-284.7	Greece	36,973	13,334	-177.3
China	9,444,000	2,677,800	-252.7	Jordan	2,700	982	-174.9
Russia	26,000	8,148	-219.1	Colombia	550,000	222,023	-147.7

The country with the greatest percentage population increase in mules was France, however it was also observed that Thailand had a 59% increase in mule population size although the population in 1997 was only 20 and the population in 2018 was 49 as such it was deemed that the population change was anecdotal. The country with the greatest percentage decrease was the USA in the number of mules. However, the greatest numerical increase in the number of mules was in Ethiopia and the greatest numerical decrease in the number of mules was in China (Table 1B).

The greatest regional percentage change in donkey population between 1997 and 2018 was in Eastern Europe with a reduction in the number of donkeys from 370,474 to 87,542. In contrast, the largest increase in the number of donkeys was found in Sub-Saharan Africa with an increase from 8,967,683 in 1997 to 20,040,426 in 2018. There were also large increase in donkey population sizes (>50%) in Northern Africa and Western Europe with Central Asia, Northern Europe, South-eastern Asia and Southern Asia showing increases between 10 and 20%. There were large decreases in Eastern Asia, Southern Europe, Western Asia, Latin America and the Caribbean, and Australia and New Zealand, although in Australia and New Zealand there was a reduction from 2000 to 1919 donkeys.

The greatest regional percentage change in mule population between 1997 and 2018 was in Eastern Europe with a reduction in the number of mules from 17,732 to 1,748, with reductions also found in Northern America, Eastern Asia, Western Asia, Southern Europe, South-eastern Asia and Northern Africa, Latin America and the Caribbean, and Central Asia. In contrast to donkey population change the largest percentage increase in mule population size between 1997 and 2018 was in Western Europe from 14,377 to 28,715, with increases in mule population size also seen in Northern Europe, Southern Asia and Sub-Saharan Africa. There was no change in the population size for mules in Australia and New Zealand as no mules have been reported between 1997 and 2018.

Further investigation of the links between donkey and mule population dynamics at the regional level shows that was a strong correlation between the reduction in the number of donkeys and mules in East Asia with (r^2^ = 0.87, *P* < 0.001). There was a weaker correlation between Western Asia, however Western Asia showed a similar trend for both donkey and mule populations reducing between 1997 and 2018 (T-test P-value <0.001). All other region showed both weak r^2^ values (< 0.5), and the changes in donkey and mule population were found to be independent (T-test P-value >0.05) indicating independent drivers of the changes in mule and donkey populations within these regions.

## Discussion

Globally there has been an increase in the number of donkeys and a decrease in the number of mules between 1997 and 2018. These result support the trend that Starkey & Starkey [2] found from 1961 to 1996 for an increase in donkey populations, and demonstrate that this trend is continuing. Whilst here have been both regional and country level reduction in the numbers of donkey and mules there have also been increases demonstrating that there is not a one size fits all approach to understand how global donkey and mule populations are changing over time with many complex socio-economic drivers at play. Some of the factors that maybe influencing the reduction in the number of working donkeys and mules may include increased movement towards agricultural mechanization as countries and agricultural systems within those countries become more economically developed [35]. Despite this, Greaub et al. [35], also identified that this can also vary at the country level with nations like Brazil becoming more industrialised, however, still relying heavily on the small scale farming sector for food security and using equids for traction. There may also be declines in donkey population size at the regional or country level due to the demand for donkey skins to produce eijao [36] which may be impacting countries further afield than just China. However, there may also be an increase in donkey breeding in other countries or regions specially to fulfill the demand for eijao via legal or illegal export [37]. Many factors may also contributing to increases in donkey population size such as increased demand for products such bricks, coal and mineral in developing countries [5, 6, 38, 39] which may be also be assisted by communities changing from using oxen to transport goods to donkeys and mules due to their greater efficacy [5].

Differing evidencing of independent tendencies in both regional and country levels trends in donkey and mules populations demonstrate a decoupling which could be attributed to a number of different reasons. For example in Europe and increased interest in farmed donkeys maybe driving the increase in population size whereas the move from mechanization in agriculture and transport maybe reducing the number of mules which are less well suited to production based farming systems [11]. Conversely, countries such as Egypt where there is a demand for mules to provide traction for industrial production whereas donkeys are less well suited to this purpose [1] we find an increase in the numbers of mules but a reduction in the number of donkeys [40].

Whilst this paper focuses on the data held by the FAO which is provided by Counties to their repository it is noteworthy that this data does not contain the resolution to understand the potential extinction of endangerment of species breeds of donkeys which can lead to the erosion of the genetic viability of these populations. Navas et al. [27], highlighted the these losses in genetic diversity can mean the loss of important functional traits. This become particular pertinent when considering the uses for different breeds of donkeys across Europe [11]. Camilo et al. [11], highlighted that there are numerous agricultural uses for donkeys with a far larger scope than just traction, for example donkeys are also used for landscape maintenance and as livestock guardians which is particularly important in Italy for which different breeds are more well suited. However, Camilo et al. [11], concluded that the move towards mechanisation and the movement of people from rural areas within the increasing size of farms from small holders to large industrial farms has driven the reduction in both overall population size and has led to the further endangerment of donkey breeds within Europe.

Starkey & Starkey’s paper concluded with suggesting that there would be an increase in the number of donkeys in sub-Saharan African countries [2]. Whilst some countries have not followed this trend there has been a 50% increase in the donkey population and a 27% increase in the mule population size in the region of sub-Saharan Africa. This suggests that the trends that were relevant from 1961 are still relevant to FAO donkey population data from 1997 to 2018.

The FAO collates the largest and most reliable data sets relating to donkey and mule populations. This data provides a valuable resource for NGO’s to target resource to areas where there are dramatic declines in populations, provides information that can be combined with other data sources to target areas where donkeys and mules population maybe at greatest risk, and allows NGO’s to identify global, regional and country level changes in population size. For NGO’s to replicate this valuable data source would be prohibitively expensive.

Whilst the FAO provides the most complete publicaly available dataset there are areas where improvements could be made specially focusing on donkey and mule populations. Improvements should focus on obvious errors in data and FAO estimates and therefore where trend data doesn’t tell the full story. An example being Sudan that shows the largest increase in donkeys since 1960s, but the reality is that there was an official figure of 578,000 in 1961 and then FAO estimates until the next official value in 2016 of 7.5 million. This manifested as a huge jump in population between 2011 and 2012. This doesn’t reflect reality, and only shows how inconsistent official data can lead to erroneous FAO estimations. Starkey & Starkey [2] showed that using the FAO donkey and mule data to understand large scale global, region and country level changes over time is appropriate the reliability may be low but broad spatial temporal trends are noteworthy for understanding changes in population size and linking these to changes in broad socio-economic changes. Where FAO population figures are derived from FAO estimates or FAO imputations, these figures may change over time. For example, before the release of the 2017 data, the FAOSTAT database indicated that the 2016 Botswana donkey population was 141,889 whereas following the release of the 2017 data, it indicated that the 2016 Botswana donkey population was 200,000. This is due to the predictive model implemented by the FAO depending on and improving when more data becomes available. Consequently, when the FAO acquire new official and unofficial data for the following year, the models will be based on a slightly different dataset, which means that the modeled values will be different to previous release but will be more accurate. Another shortcoming of the FAO dataset is that the number of live donkeys and mules represents all genus regardless of age, sex, breed or purpose raised. This introduces issues when trying to accurately establish the number of equids in working or farmed environments with the two population having very different welfare concerns making it difficult for NGO’s to understand where and which equids are in the greatest need [4, 38, 41, 42].

Despite the broad global regional and country levels changes in donkey and mule populations that can be derived from the FAO data sets the information provided by the FAO does not consider the detailed nuances within specific breeds, sex, or type of role that the equids play in society (i.e working vs companion animal vs wild). As such using the FAO data it is difficult to draw conclusions for the extinction or endangerment of specific breeds or how certain industries are affecting changes in population size and distribution. However there is a growing body of research relating to these areas for example there is evidence for the declines in population in Europe may be related to the loss of certain breeds of donkeys [11, 27].

In order to support the FAO more effort needs to be made at the country level for promoting the reporting of figures, which can then disseminate this information via FAOSTAT. Currently a large number of countries including Nepal, United Kingdom and Kenya do not report donkey or mule population sizes to the FAO. In the United Kingdom separate records are held in a Central Equine Database that is behind a paywall and requires strong working relationships to gain access. In contrast, Nepal reports no information and no information is held by the central government and as such it is difficult to understand the magnitude of the welfare crisis that effect equids working in Nepalese brick kilns [38, 39]. Where countries do report information to the FAO there may be some discrepancies [2]. For example, during a recent African Horse Sickness outbreak in Thailand, local animal welfare charities reported the total population of equids (horses, mules and donkeys) as ~12,000 based on recent reports by the Department for livestock development (*per comms* Dr Siraya Chunekamrai). Whilst the FAO indicates that, there is a total equid population of 6,148 suggesting a shortfall of ~6,000 equids between figures reported locally and those held by the FAO.

To improve the quality of data held by the FAO, NGO’s can support the FAO’s work by discussing with partner organization and government bodies at the country level about reporting strategies and support information transfer to the FAO in a timely manner. By encouraging reporting to a centralized resource such as FAOSTAT, there would be an improvement in the reliability of the information held by the FAO. Which would in turn benefit governments, NGO’s and other stakeholders needing this information to make informed decisions about where to target resources to support equids in the greatest need.

## Conclusions

Globally donkey populations have been steadily raising since 1961 and as shown by Starkey & Starkey [2] increased between 1961 and 1996, with this study showing that this trend has continued until 2018. There has however been a steady decline in the number of mules from 1997 to 2018. This paper demonstrates that there is not a one size fits all explanation for these changes in population dynamics and there maybe regional, country or even local level drivers at play. This paper therefore makes an important contribution to the literature through understanding how these trends are developing and understand what emerging drivers could be influencing these temporal changes in populations. What is clear from investigating the global population estimates for donkeys is that there are complex underlying socio-economic drivers affecting donkey populations within each country, thus it is difficult to attribute any change in population to a single factor. Where we do see a dramatic decline in donkey population size (such as in China), there is no evidence in the FAO data that there is a direct link to the skin trade.

The FAO livestock population estimates for donkeys and mules are the most complete and consistent source of population data. They are easily accessible and come from an organisation that is actively involved in developing statistical knowledge and understanding worldwide. NGO’s should focus efforts on promoting the reporting of information to the FAO in order to strengthen the reliability of the information held by the FAO to be used in the future to identify populations at the global, regional and country level that are in the greatest need of support.
